# Fatal Mycotic Aneurysm of the Basilar Artery Caused by *Aspergillus fumigatus* in a Patient with Pituitary Adenoma and Meningitis

**DOI:** 10.3389/fmed.2017.00113

**Published:** 2017-07-18

**Authors:** Martin Winterholler, Roland Coras, Walter Geißdörfer, Rudolf Rammensee, Philipp Gölitz, Christian Bogdan, Roland Lang

**Affiliations:** ^1^Neurologische Klinik, Sana-Krankenhaus Rummelsberg, Schwarzenbruck, Germany; ^2^Department of Neuropathology, Universitätsklinikum Erlangen, Friedrich-Alexander-Universität Erlangen-Nürnberg, Erlangen, Germany; ^3^Mikrobiologisches Institut—Klinische Mikrobiologie, Immunologie und Hygiene, Universitätsklinikum Erlangen, Friedrich-Alexander-Universität (FAU) Erlangen-Nürnberg, Erlangen, Germany; ^4^Department of Neurosurgery, Universitätsklinikum Erlangen, Friedrich-Alexander-Universität (FAU) Erlangen-Nürnberg, Erlangen, Germany; ^5^Department of Neuroradiology, Universitätsklinikum Erlangen, Friedrich-Alexander-Universität (FAU) Erlangen-Nürnberg, Erlangen, Germany

**Keywords:** fungal infection, meningitis, galactomannan antigen, PCR aspergillosis, β-d-glucan antigen

## Abstract

Fungal infections of the central nervous system (CNS) frequently occur in immunosuppressed patients. Here, we describe the case of an immunocompetent 64-year-old man who presented with diplopia, right-sided hemiparesis, and a mild headache after cleaning and replacing nesting boxes of wild birds during the preceding months. Lumbar puncture revealed pleocytosis, elevated protein, and lactate levels in the cerebrospinal fluid (CSF). Initial imaging showed ischemia in the left thalamus and an enlargement of the sellar region. Antibiotic treatment and corticosteroids led to an initial improvement but was followed by rapid deterioration. Antibiotic treatment was modified and antifungal therapy was added. Eighteen days after admission, the patient died from a subarachnoid hemorrhage resulting from the rupture of a fusiform aneurysm of the basilar artery. Microbiological culture of CSF was negative, but a positive galactomannan assay suggested fungal infection which was corroborated by detection of *Aspergillus fumigatus* DNA in pan-fungal PCR and sequencing. The presence of septated hyphae in the wall of the basilar artery confirmed the diagnosis of a mycotic aneurysm caused by hyphomycetal infection. In addition, brain autopsy revealed the presence of an invasive adrenocorticotrophic hormone-producing pituitary adenoma with arrosion of the sellar bone. This process and its invasiveness likely facilitated the spread of the fungal pathogen from the sphenoid sinus to the dura mater and finally led to cerebral angioinvasion. Our case demonstrates the challenge to timely diagnose and effectively treat aspergillosis as a cause of CNS infection also in apparently immunocompetent patients. The potential of assays detecting fungal antigens and of PCR to facilitate a timely diagnosis is discussed.

## Background

Infections of the central nervous system (CNS) are characterized by a combination of neurological symptoms (headaches, nuchal rigidity, focal signs) and inflammatory signs such as fever. In addition to clinical examination and radiologic imaging, prompt laboratory analysis of cerebrospinal fluid (CSF) is decisive in the differential diagnosis of CNS infection. The number and type of leukocytes, protein content, and the levels of glucose and lactate allow to obtain an initial diagnosis of meningitis and to judge the likeliness of viral versus bacterial infections. Microscopy of Gram-stained CSF specimens and antigen tests for bacterial cell wall polysaccharides often allow the rapid identification of typical bacteria causing meningitis such as *Streptococcus pneumoniae, Neisseria meningitidis*, or *Haemophilus influenzae*. Detection of bacterial pathogens by culture increases the sensitivity of microbiological diagnostics and can be complemented by PCR-based detection of bacterial DNA using 16S rRNA-gene specific primers and sequencing, which is especially important in patients already receiving antibiotic treatment. In contrast, fungal CNS infections are less frequent and much more difficult to diagnose. They typically affect immunocompromised hosts, with cryptococcosis in AIDS patients as most important example. Aspergillosis of the CNS is much more frequent in hosts with severe immunosuppression, but it is estimated that 50% of meningitis cases due to *Aspergillus* spp. occur in patients without apparent immunosuppression (1). CNS aspergillosis can develop by direct extension of infections of the ear, orbital, or paranasal sinuses, by iatrogenic inoculation during surgery or spinal anesthesia, or through the hematogenous route in patients with pulmonary aspergillosis. The prognosis of CNS aspergillosis is poor with an overall fatality rate of around 70%, which can reach close to 100% in patients with hematological malignancies. In many patients, the identification of *Aspergillus* spp. as causal agent is achieved only retrospectively after the death of the patient, which underlines the diagnostic challenges associated with this condition. Importantly, early diagnosis of aspergillosis leads to increased survival rates. We present the case of a 64-year-old man who developed a fatal mycotic aneurysm of the basilary artery due to unrecognized infection with *Aspergillus fumigatus*.

## Case Report

### Clinical Course and Initial Diagnostic and Therapeutic Management

A 64-year-old man presented to the emergency department with diplopia, vertigo, unstable gait, and dysarthria. He reported to have had headaches during the preceding 3 weeks. Apart from a type II diabetes mellitus, which was reasonably controlled by metformin (HbA1c 6.4%), his medical history was unremarkable. Upon physical examination, the patient had a slurred speech, slight right-sided brachiofacial hemiparesis, and partial oculomotor paresis on the left, but no nuchal rigidity. Body temperature was 38.1°C.

On the day of admission, a cranial CT was performed and showed a small hypodensity in the left thalamic region, compatible with focal ischemia. Subsequent lumbar puncture and CSF analysis revealed a mixed pleocytosis (700 cells/μL), elevated protein and lactate (3.5 mmol/L), and slightly reduced glucose (53 mg/dL). No microorganisms were detected in the Gram stain of the CSF. Latex agglutination tests for cell wall polysaccharides of typical bacteria causing meningitis (*S. pneumoniae, N. meningitidis*, group B streptococci, *H. influenzae*) were all negative. Empiric therapy for meningitis was started with a combination of ceftriaxone, ampicillin/sulbactam, and aciclovir. The initial and repeated CSF cultures taken on days 4 and 10 after hospitalization remained sterile, and PCR for 16S bacterial rRNA genes as well as specific PCRs for *Tropheryma whipplei, Mycobacterium tuberculosis*, non-tuberculous mycobacteria, and the herpes viruses HSV and VZV were all negative. No evidence for infection with *Borrelia burgdorferi, Treponema pallidum*, FSME, or HIV was found by serology.

**Figure 1:**
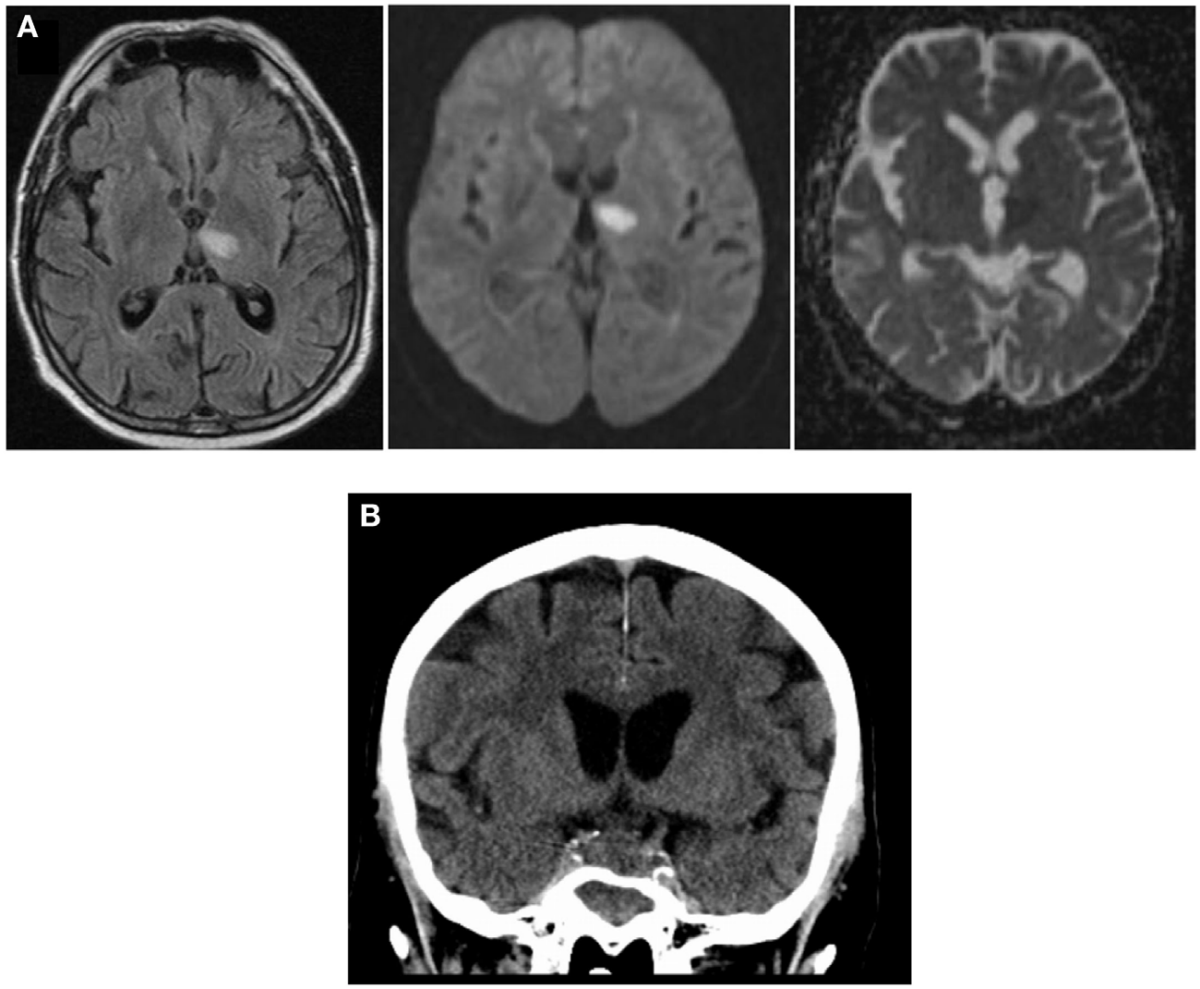
Imaging during course of disease: **(A)** NMR imaging on day 3 after admission shows hyperintensity in FLAIR sequence (left), high degree of diffusion-positivity (DWI, middle), and lack of shine-through effect in ADC (right), indicating acute thalamic infarction. **(B)** cCT scan (day 10) showing increased sellar process.

NMR imaging on day 3 revealed infarction in the left thalamus, based on diffusion-weighted imaging and hyperintensity in the FLAIR sequence (Figure 1A), as well as an enlargement of the sellar region consistent with an adenoma of the pituitary gland. Despite an initial clinical response to the treatment with antibiotics and corticosteroids, which was accompanied by a drop of the CSF cell count to 280/μL on day 3, the patient continued to have fever. As he deteriorated clinically and the pleocytosis of the CSF persisted, the antibiotic regimen was changed to a combination of ampicillin, levofloxacin, and doxcycline because anamnestic exposure to sheep and excretions of wild birds during cleaning of nesting boxes suggested the possibility of a zoonotic pathogen. However, serological tests for antibodies to *Coxiella burnetii, Rickettsia* spp., *Brucella* spp., *Mycoplasma pneumoniae, Leptospira* spp., and *Francisella tularensis* were all negative; *Toxoplasma gondii*- and *Chlamydia*-specific antibodies were detected at low titers, consistent with past infections. Analysis of the CSF for cryptococcal antigen was repeatedly negative.

**Figure 2:**
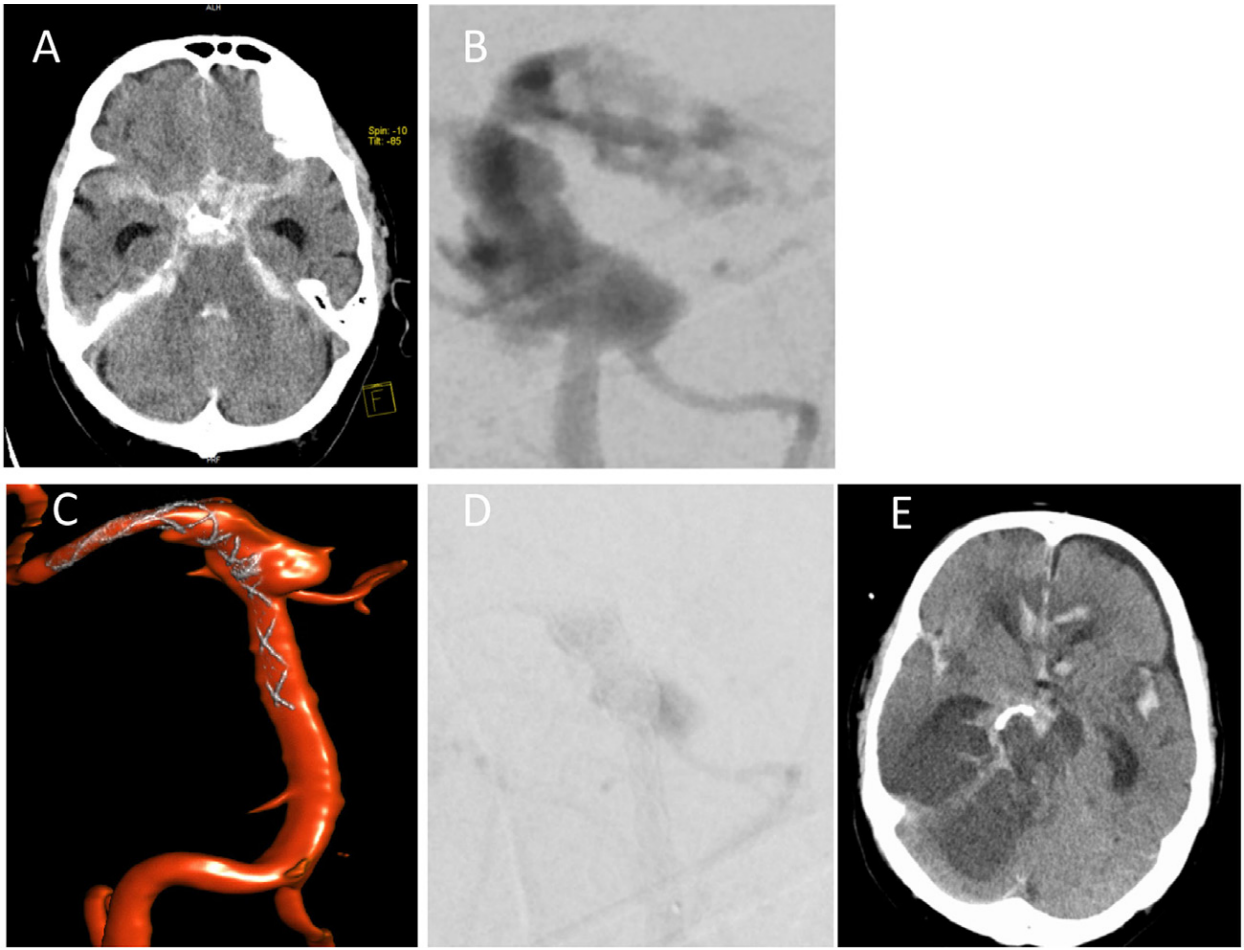
Subarachnoidal hemorrhage from *A. basilaris*. **(A)** cCT on day 12 scan showing hemorrhage. **(B,C)** Digital subtraction angiography with intervention showing active bleeding **(B)** and insertion of flow diverting stent **(C)**. **(D)** Late arterial phase of DSA indicating starting thrombosis immediately after flow diverter implantation. **(E)** Follow-up cCT on day 17 shows demarcation of ischemic areas.

On day 8, NMR imaging was repeated and suggested vasculitis of the basilar artery with a small infarction of the brain stem. The cCT scan obtained on day 10 revealed sinusitis sphenoidalis and an increase in the space-occupying sellar process was observed, leading us to consider a fungal infection (Figure 1B). Therefore, additional treatment with the antimycotic agent voriconazole was initiated (200 mg every 12 h). On day 12 after initial hospitalization, the patient was transferred to the neurosurgical department of the university hospital to obtain a tissue biopsy and histology of the sellar process. Within 2 h after transfer, the patient deteriorated dramatically, developed a respiratory arrest and absent pupillary reflexes. Emergency cCT showed a massive subarachnoidal hemorrhage (Figure 2A), caused by active bleeding from a fusiform aneurysm of the basilar artery tip as revealed by digital subtraction angiography (Figure 2B). Bleeding could be stopped by insertion of a flow diverting stent (Figures 2C,D). However, the patient did not improve clinically during the following days but was comatose without reaction to nociceptive stimuli yet with intact corneal reflexes. The EEG showed a burst-suppression pattern. On day 17, large demarcated infarction areas were seen by NMR in the mesencephalon, the cerebellum, and brain areas supplied by the *A. cerebri* anterior and posterior (Figure 2E). Given the poor prognosis at this stage, a decision was made to limit therapy after consultation with the family of the patient. The patient died on day 18 after admission.

### Diagnosis of Fungal Infection

Although cultures of repeated CSF samples did not yield growth of bacteria or fungi, the development of a presumptive mycotic aneurysm of the basilar artery despite broad and high-dose antibiotic therapy, along with the radiologic suspicion for a possible infectious process in the sellar region, prompted us to re-consider a fungal infection in this patient. Therefore, remaining CSF samples from days 4, 10, and 13 after admission were analyzed for the presence of *Aspergillus* galactomannan antigen using the Platelia™ assay (Bio-Rad, Marnes la Coquette, France) (Table 1). The index values were positive in all three samples (4.2, 10.2, and 1.4, respectively, cutoff 0.5); serum was only tested for *Aspergillus* galactomannan on day 13 and was negative. CSF samples taken at day 4 and day 10 were weakly positive for fungal 5S–28S rRNA spacer DNA using PCR amplification and identified as derived from *A. fumigatus* by sequencing (Table 1).

**Table 1:** Summary of laboratory findings for fungal cultures, PCR, and galactomannan assays.

Sample	Test	Days after hospitalization							Autopsy
		0	3	4	8	10	12	13	
Cerebrospinal fluid (CSF)	Platelia *Aspergillus* antigen			4.2		10.2		1.4	
Serum	Platelia *Aspergillus* antigen							0.1	
CSF	Internal transcribed spacer 2 PCR	(−)		(+)		(+)			(−)
CSF	Fungal culture	(−)		(−)		(−)			
Brain tissue	Fungal culture								+ *A. fumigatus*

**Figure 3:**
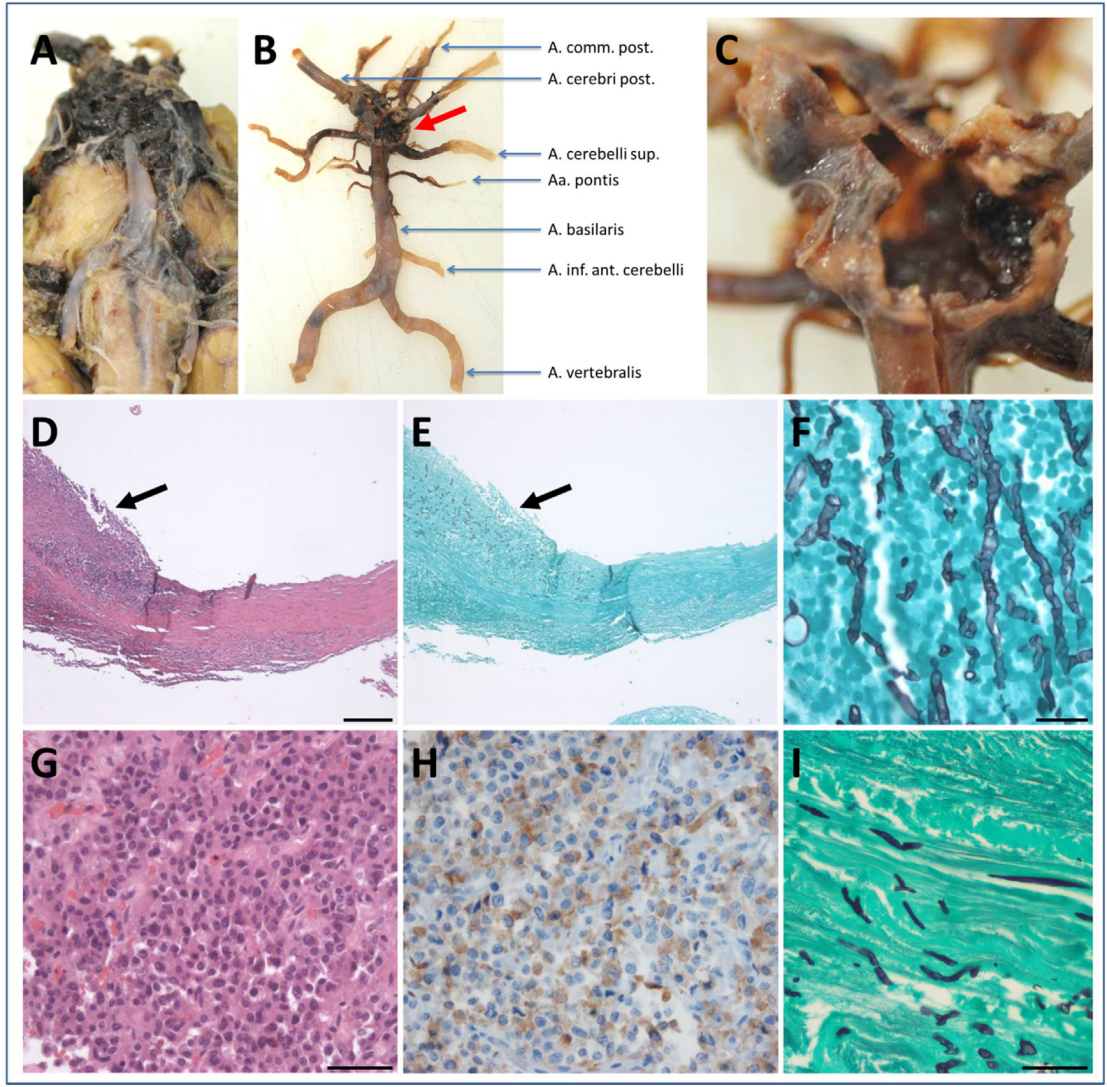
Neuropathological findings. Macroscopic findings **(A–C)**: **(A)** subarachnoid hemorrhage within the basal cisterns coating the midbrain and pons and extending to the cerebellopontine angle. **(B)** Macroscopic view of the vertebrobasilar vascular supply after preparation of the hematoma clearly showing an aneurysm at the basilar tip (red arrow). Higher magnification in **(C)** demonstrates the ruptured aneurysm. Microscopic findings **(D–I)**: **(D)** hematoxylin & eosin (H&E) staining of the basilar aneurysm showing thickening and splitting of the wall due to intramural inflammatory infiltrates (arrow). **(E)** Same area as depicted in **(D)** the presence of hyphal structures in close association to the inflammatory infiltrates confirms the diagnosis of a mycotic aneurysm (Grocott histochemistry). **(F)** Higher magnification of **(E)** (Grocott histochemistry). **(G)** A pituitary adenoma visible in the sellar region (H&E staining) with immunohistochemical expression of adrenocorticotrophic hormone **(H)** proving the diagnosis of a corticotroph adenoma. **(I)** Hyphal structures within the dura adjacent to the corticotroph adenoma (Grocott histochemistry). Scale bar in **(D)** 200 µm, applies also for **(E)**. Scale bar in **(F)** 20 µm. Scale bar in **(G)** 50 µm, applies also for **(H)**. Scale bar in **(I)** 100 µm.

Brain autopsy revealed generalized brain edema with uncal/transtentorial as well as tonsillar/cerebellar herniation. At the sites of herniation, there were foci of necrosis and hemorrhage including the midbrain and pons region. There was prominent subarachnoid hemorrhage within the basal cisterns and intraventricular extension of the hemorrhage. In addition, widespread hypoxic–ischemic damage in supra as well as infratentorial areas was observed. The cause of death was a rupture of an aneurysm of the basilar artery (Figures 3A–C). Microscopic analysis of the aneurysmatic vessel wall revealed inflammation and foci of hyphal structures with septae and dichotomous ramifications, which were best visualized by Grocott histochemical staining (Figures 3D–F), arguing for a mycotic aneurysm due to a hyphomycetal infection. The sellar region showed a pituitary adenoma with immunohistochemical expression of adrenocorticotrophic hormone (ACTH) in terms of a corticotroph adenoma (Figures 3G,H) with invasiveness concerning the adjacent bone and dura. There was also invasion of hyphae into the dura surrounding the sphenoid sinus (Figure 3I). Furthermore, one of the autopsy samples (taken from the right thalamus) was culture-positive for *A. fumigatus* (Table 1). Thus, invasion and destruction of adjacent tissue by the pituitary adenoma may have facilitated the intracranial invasion of *A. fumigatus* and subsequent vasotropic infection of the basilar artery.

## Discussion

### CNS Aspergillosis in Immunocompetent and Immunosuppressed Patients: Risk Factors and Predilections

Invasive aspergillosis is a well-recognized problem in severely immunosuppressed patients and frequently (i.e., in 10–20% of disseminated aspergillosis cases) affects the brain with high lethality rates. The main causes of immunosuppression predisposing to the development of disseminated aspergillosis are HIV infections with low CD4^+^ T cell numbers (AIDS), solid organ or bone marrow transplantation, extended treatment with steroids, and diabetes. However, while much rarer in immunocompetent individuals, CNS aspergillosis also occurs in the absence of overt immunosuppression. In a recent literature review of 93 cases of *Aspergillus* meningitis, Antinori et al. found that more than 50% of all patients did not have a history of immunosuppression. In immunocompetent hosts, CNS aspergillosis can result from the spreading and direct invasion of localized infection of the sinuses, the orbita, or the ear (1–3). Furthermore, iatrogenic infections following spinal anesthesia, neurosurgery, or epidural steroid injections account for a significant fraction of CNS aspergillosis in the immunocompetent host (1).

In the patient reported here, no clinical immunosuppression was evident at presentation: leukocyte counts were normal, and the diabetes mellitus type 2 was sufficiently well controlled based on an HbA1c level of 6.4%. In addition, the patient had not received steroid therapy before admission, and the duration of the glucocorticoid treatment after diagnosis of meningitis was only 5 days. The discovery of an ACTH-producing adenoma of the pituitary gland in the postmortem neuropathological analysis raised the possibility of an underlying hypercortisolism, which would cause higher susceptibility to invasive aspergillosis (4). In fact, a case of *Aspergillus* infection of the sinus sphenoidalis infiltrating the neurohypophyseal tissue in a patient with Cushing’s disease due to an ACTH-producing adenoma–gangliocytoma of the pituitary has recently been described (5). However, no clinical signs of Cushing’s disease were seen in our patient, arguing against hypercortisolism as the driving force in this patient. On the other hand, since cortisol levels in the serum or excretion in the urine were not determined, this scenario cannot be excluded. A previous NMR imaging study suggested the spreading of *A. fumigatus* from infected paranasal sinuses per continuitatem into the CNS (6). Based on the demonstration of *Aspergillus* hyphae in the dura mater adjacent to the sphenoid sinus (Figure 3E), it is conceivable that in our patient the fungus took the same route of invasion. Due to the ubiquitous presence of *Aspergillus* spores in the environment, the source of infection can be manifold. Our patient had repeatedly cleaned and replaced nesting boxes of wild birds in the months preceding his illness and therefore may have been exposed to high doses of *A. fumigatus* conidia during this work.

### Mycotic Aneurysm: Angiotropism of *Aspergillus*

Infiltration of blood vessels is a well-known characteristic of *Aspergillus* infection and can lead to thrombosis, formation of mycotic aneurysms, and bleeding. In CNS aspergillosis, destruction of arteries is a common complication leading to subarachnoid hemorrhage. A review of the literature on *Aspergillus* meningitis reported that 12 of 49 patients subjected to autopsy had a mycotic aneurysm of the internal carotid or the basilar artery (1). Our patient had a fusiform aneurysm of the basilar artery; evaluation of the entire cerebral circulation by DSA demonstrated no further aneurysm and especially no vessel wall irregularity of the cavernous segment of both internal carotid arteries. As in the case reported here, fungal aneurysms are usually fusiform (7). After interdisciplinary case discussion, implanting a flow diverting stent was chosen as therapy approach to treat this acutely ruptured fusiform aneurysm of basilar artery tip, presenting active bleeding during diagnostic angiography. Certainly, this procedure harbors the risk of perforator occlusion and subsequent perforator infarction in 14–25% (8, 9). Implanting non-flow diverting, braided self-expandable low-profile stents (e.g., the LEO Baby stent, Balt Extrusion, Montmorency, France) could be considered an alternative treatment approach, possibly using two stents in telescopic dual-stent configuration to increase flow diversion effect but still to reduce the risk of acute thrombosis of the parent artery. Aspergillosis tends to occur in perforating arteries of the brain (10) which supply the thalami and basal nuclei. The distribution of ischemic infarction early in the clinical course of this patient (Figure 1A) is therefore consistent with a causal role for angiotropic aspergillosis, although at autopsy no hyphae were detected in the ischemic areas.

### Diagnostic Challenges in CNS Aspergillosis

The rapid diagnosis of *Aspergillus* as a cause of CNS infection is a challenging task. This has been impressively documented by the review of 92 cases of CNS aspergillosis, of which only around 56% were diagnosed during life (1). First, in apparently immunocompetent patients, fungal infections are often only considered after the failure of the initial standard antibiotic therapy for common bacterial causes of meningitis. In this regard, the presence of imaging findings consistent with aspergillosis, such as infarction (especially in the basal nuclei and/or thalami), subarachnoid hemorrhage, abscess formation with ring enhancement, or infection of the paranasal sinuses, is suspicious for cerebral aspergillosis (6, 10). Second, a main obstacle has been the poor sensitivity of culture-based diagnostics, which has been estimated to be only around 30%. Increasing the volume of CSF used for culture and repeated sampling have been suggested to increase the rate of detection (1). This low sensitivity is illustrated in the case presented here, since despite repeated lumbar punctures during the course of disease, *A. fumigatus* was only found by culture from a brain tissue sample obtained during autopsy.

The use of alternative, non-culture-based methods for detection of *Aspergillus* spp. show some promise to improve this diagnostic dilemma. The detection of fungal cell wall antigens by the galactomannan test (Platelia™ Antigen EIA) is well established for the use in serum to detect invasive aspergillosis and monitor treatment responses and is recommended by the manufacturer for use with bronchoalveolar lavage fluid. While a cutoff value for the use with CSF has not been established and the test is not designated for this use by the manufacturer, it had a sensitivity of 87% in the 15 patients reported in the literature review by Antinori et al. (1). The galactomannan assay showed similar good sensitivity in reports with small numbers of patients (11–13). The galactomannan measurements of several CSF samples from our patient support the usefulness of this assay for diagnosis of CNS *Aspergillus* infection. Detection of β-d-glucan (BDG), which is also present in the *Aspergillus* cell wall, may be another approach to increase the sensitivity of laboratory based diagnostics. BDG assays are well established for the use in serum, where they have high sensitivity but lower specificity to detect systemic aspergillosis (14). There are only limited reports of their application to the testing of CSF samples, with encouraging results (15, 16). Thus, while the existing data are based on small numbers of patients, the use of galactomannan and/or BDG assays on CSF samples may significantly shorten the time to diagnosis of CNS aspergillosis.

PCR tests specifically detecting *Aspergillus* DNA sequences or amplification of pan-fungal internal transcribed spacer 2 (ITS 2) sequences followed by sequencing (used in this case) represent another non-culture-based diagnostic approach. In our case, two of four CSF samples were weakly positive for *A. fumigatus* DNA in the pan-fungal PCR assay (Table 1). To date, only few studies have investigated the usefulness of PCR for *Aspergillus* detection from CSF, with somewhat controversial results regarding the sensitivity of this approach (17–19). Given the low sensitivity of detection by culture, PCR methods could complement antigen tests to further increase the sensitivity and specificity of *Aspergillus* diagnostic tools (20).

## Ethics Statement

This case report was submitted to the Ethics Committee of the Friedrich-Alexander Universität Erlangen-Nürnberg and a waiver of informed consent was obtained.

## Author Contributions

MW and RR treated the patient. PG analyzed neuroradiological images. RC performed neuropathological analysis. WG, CB and RL performed micobiological diagnostics. RL wrote the manuscript with input from all other authors.

## Conflict of Interest Statement

The authors declare that the research was conducted in the absence of any commercial or financial relationships that could be construed as a potential conflict of interest.
